# Medical Evidence:—The Colney Hatch Case

**Published:** 1861-01

**Authors:** 


### Art. VIII.—MEDICAL EVIDENCE:—THE COLNEY HATCH CASE.

On the 12th of May last a lunatic, named William Swift, died suddenly in the Colney Hatch Asylum. The attendants stated that shortly before his death Swift staggered and fell in a convulsion which was regarded by them as an ordinary epileptic fit. From this the patient quickly recovered in a great measure, said that he was better, and asked for his supper, part of which he ate, but becoming suddenly worse, the medical superintendent of the male department, Mr. D. F. Tyerman, was sent for, but before he reached the ward in which Swift was confined, the latter had died. On the 14th, two days after death, the body was examined in presence of Mr. Tyerman. Several slight marks were observed on the neck and face of the patient. On the left side of the neck was a thin mark of a yellowish colour; on the right side of the neck, and also on the right side of the face and lobe of the right ear were slight marks of a bluish colour. On the abdomen, to the right of the umbilicus, was an extensive bruise. The marks on the neck were probably to be accounted for, and the bruise upon the abdomen was known to have been occasioned, by a serious struggle which Swift had had with one of the attendants three days prior to his death. Sundry marks upon the left wrist had been caused by the use of wrist-locks previous to the patient's admission into Colney Hatch.

No other indications of injury were discovered by the inspection of the body externally.

On the chest being opened it was found that the sternum had been fractured across the centre; that on the right side of the thorax, the 4th, 5th, 6th, 7th, 8th and 9th ribs had been fractured; on the left, the 4th, 7th, 8th, 9th and 10th. In each of the injured ribs the fracture existed a short distance from the cartilage, but in neither ribs nor sternum was there any displacement of the fractured parts. Beneath the right costal pleura there was ecchymosis in the vicinity of the fractures; there was a layer of blood in the anterior mediastinum; and there were about three ounces of sanguineous fluid in the left pleural cavity. The lungs were generally healthy and moderately collapsed; in the apex of the right lung cretefied tubercle was found. The heart was not well contracted, and both sides were empty.

On examining the abdominal cavity a large quantity of partially coagulated blood was discovered within it, and on the posterior surface of the left lobe of the liver there was found a laceration, two inches in length, but "not deep." A slight superficial laceration also existed on the surface of the right lobe.

The brain was examined, but no morbid appearances of moment, as bearing upon the cause of death, were noted.

There could be no doubt that the grave and extensive injuries so unexpectedly discovered in the chest and abdomen had caused the sudden death of the unfortunate lunatic, and the strictest inquiries were made in the ward where Swift had been confined at the period immediately preceding, and at the time of his death, to ascertain if he had been subjected to any violence on the part of the attendants. Nothing, however, was detected which yielded any confirmation of this first and most natural surmise. But it was known that on the evening of the 9th, about seventy-two hours before his sudden death, Swift had had, in another ward, a severe struggle with one of the attendants, a very powerful man. Swift, at the time in question, had retired to his bedroom for the night. There was then but one attendant on duty in the ward. Swift became noisy, knocking loudly at the door of his room, and crying out that he wished to see the Queen. In order to pacify him the attendant went to his room, but no sooner had the man entered the doorway than Swift struck him violently on the eye, and seized him by the neckerchief, making use of an expression which showed that he had been watching his opportunity. A desperate struggle now ensued, and both fell together upon the floor of the bedroom, the lunatic still retaining firm hold of the attendant's throat, who was barely enabled to call for aid to a tranquil patient engaged in an adjacent scullery. Through the instrumentality of this patient assistance arrived just in time to avert fatal consequences to the prostrated attendant. He was found still held by the throat in the grasp of Swift, the blood was oozing from his nose and mouth, the face was purple, and he was slightly insensible. Swift, almost exhausted by his efforts, released the attendant when aid arrived, and retired to his bed; and when again visited by both attendants, he bid them good-night, making no complaint of pain or injury. On the following morning the circumstances of the assault were reported to the medical superintendent, and the attendant who had been attacked stated that in his efforts to free himself he had struck Swift violently on the abdomen, which was bruised in consequence. The bruise thus occasioned was the one recorded in the notes of the *post-mortem* examination as being found to the right of the navel.

Could it be that the fractures of the ribs, and sternum, and lacerations of the liver, found in Swift's body after death, were caused by the struggle between him and the attendant on the

9th of May? Mr. Tyerman thought that this probably might have been the case; no evidence whatever being forthcoming, notwithstanding reiterated and most diligent research to show the likelihood of the injuries having been inflicted at any other time.

Swift was fifty-four years of age, a labourer, and had been admitted into Colney Hatch on the 24th February, 1860. It was then stated that he was suicidally disposed, very violent towards all who approached him, and that he had attempted to strangle the master of the Aldgate Workhouse. He was the subject of incipient general paralysis, the articulation being imperfect, the mind confused, and the general health much injured. His arms were marked with the pressure of wrist-locks, and the left wrist was much galled and blistered. He was first placed in the infirmary, and under the use of a nutritious diet and wine his health considerably improved. On the 8th of March, he was removed from the infirmary into one of the wards. Whilst in this division of the establishment his health continued to improve, and the paralytic affection greatly diminished. His delusions were freely expressed. He thought that he was the strongest man in the world, that he could infuse strength into others by blowing into their mouths and ears, and that he had killed several hundreds of Catholics. Such excitement as he manifested was usually associated with cheerfulness, and he was gratified when any mark of attention was shown to him. On the 9th of May he assaulted one of the attendants in the manner already described, and on the 10th he was removed, for greater safety, into another ward to which three attendants were attached, and in which there were special arrangements for the management of dangerous patients. He made no complaint of suffering, was somewhat excited, and admitted his intention to kill the attendant he had attacked. He moved about and took his meals as usual, and showed no signs of indisposition. On the day following, the 11th, he continued in an excited state, spoke freely of his power to infuse strength into others, and alluded in a cheerful boasting way to "his bruise," referring to the bruise on the abdomen. On this day an emetic was prescribed for him with a view of soothing the agitation, and he had also a warm bath as was customary on the Friday with all the patients in his ward. The appetite and movements were not affected. On the 12th he continued in the same state until evening, when he died suddenly, as already related.

From the history of the case, Mr. Tyerman considered that it was not improbable that the patient's sensibility to pain had been considerably diminished (a common occurrence in mania accompanied with paralytic symptoms); and that this loss of sensibility had masked the symptoms of injury to the walls of the chest and v er. Further, believing the death to have been immediately occasioned by the hæmorrhage from the liver, he expressed the opinion, which he believed to be fully supported by several cases on record, that it was not necessary to suppose that the fatal hæmorrhage should have happened contemporaneously with the infliction of the injury, and that even an interval of seventy-two hours between the fatal effusion and the laceration of the organ was not inconsistent with surgical experience of ruptures of the liver.

Mr. Tyerman concluded, therefore, that, evidence to the contrary being altogether lacking, the whole of the injuries to Swift might be reasonably accounted for by the desperate struggle and the heavy falls consequent thereupon, which occurred three days before his death.

On the 17th of May a coroner's inquest was held on the body of the deceased lunatic, and an open verdict was returned.

Subsequently, the case was reported to the Commissioners in Lunacy, and they instituted a special inquiry into the circumstances of Swift's death. In the progress of this inquiry three or four of the lunatics who had been confined in the same ward with Swift at the time of the fatal event, stated for the first time that he had been subjected to great violence by two of the attendants, Slater and Vivian, shortly before his death. (The principal witness made his first statement on the 2nd of August.) Upon this information the Commissioners determined to take legal proceedings against these men, and on the 19th of September they were arraigned at the Central Criminal Court on a charge of manslaughter. Two lunatics only were brought forward in support of the charge, Samuel Clark and William Varney. Both men asserted that Swift had been treated with brutal violence by the prisoners shortly before his death. Clark stated that on the afternoon of the 12th (the day of death) Swift had been seized by Slater and Vivian, thrown upon the ground, dragged to a padded room, pounded with the fists, stamped upon with both feet, and also knelt upon. Varney asserted that Swift had been thrown down and dragged to his room, by the prisoners, on the afternoon of the 11th, and that he heard sounds of scuffling from the room, and the voice of Swift exclaiming, "Oh! Oh, Lord!" This witness on his preliminary examination before the magistrates had said that this scene had occurred on the 10th. Clark's account also differed in one or two important points from the evidence he had given before the magistrates. Varney, moreover, stated that it was usual for the attendants to take patients into the padded room and there "kick them about." There had been several severe cases, he asserted, in his ward, of late, the patients being quite sore after the violence. Previous to his admission into Colney Hatch, Varney had been confined at Hanwell, and he spoke of the brutal violence to which he had frequently been subjected there on the part of the attendants, the doctors laughing at him when he complained. It is evident, indeed, that this witness was subject to delusions on the subject of violence.

In addition to the testimony of these two witnesses, medical evidence was also tendered to show that it was "impossible" that the injuries from which Swift had succumbed had been inflicted long before death. Mr. Luke, Vice-President and formerly President of the Royal College of Surgeons, Senior Surgeon to the London Hospital, and Surgeon to St. Luke's Hospital for Lunatics; Mr. Partridge, one of the Surgeons to King's College Hospital, and Professor of Anatomy in King's College; and Mr. Barnard Holt, the Senior Surgeon to the Westminster Hospital, gave evidence to this effect.

According to the report of the trial in the *Times*, Mr. Partridge "deposed that he had been in court and had heard all the evidence that had been adduced, and particularly that portion of it relating to the injuries the deceased was alleged to have received. His opinion was, that it was impossible for such injuries to have been inflicted on the Wednesday without the deceased exhibiting marked symptoms of having received them during the interval between that time and his death. The consequence of so many ribs being broken would be to most materially affect the respiration, and the patient could not breathe regularly, but would do so with great difficulty, and in such a manner as must immediately attract attention. According to his judgment, the injuries that were the cause of death had been inflicted not more than two hours before. He did not think that the fact of the deceased being a lunatic would have made any difference in this case as to the consequences of the injuries that had been received. Lunatics who had met with an accident, or who had received injury from any cause whatever, would, no doubt, act very differently from sane persons, and they might under some circumstances exhibit an indifference to pain; but in this case the chest would have entirely lost the support of the ribs, and the effect upon the respiration, in his opinion, must have taken place, and the patient could not have had any control over it.

"In cross-examination Mr. Partridge said that, if so much violence had been used towards the deceased as had been deposed to by the two witnesses Clark and Varney, he should certainly expect there would be some external marks upon the body of the deceased.

"Mr. Luke gave evidence similar to that given by Mr. Partridge. He also said he agreed with that gentleman as to the possibility of a lunatic evincing an indifference to or concealment of pain in some cases, but that this could not be done when a patient had received such serious injuries as were exhibited in this case, and that the difficulty of breathing must have been apparent.

"Mr. Barnard Holt, one of the surgeons to Westminster Hospital, gave similar evidence, and this closed the case for the prosecution."

At the preliminary examination of the prisoners before the magistrates, Mr. Luke is reported to have said (*Times*, August 11th):—

"I have had considerable experience as a surgeon, particularly with reference to the treatment of lunatics. I see all the patients as admitted at St. Luke's. I have heard the evidence in this case, especially the medical evidence with regard to the *post-mortem* examination. In my opinion the deceased man Swift died from the general injuries described, which, in my judgment, must have been inflicted at one and the same time. I should think that death followed speedily on those injuries. I believe it to be impossible that a person who had received such injuries could walk about in apparently good health, his constitution exhibiting no derangement, and his pulse giving no indication that he had been injured. The fact of the person injured being a lunatic would not make any material difference. The injuries might be produced by external pressure without producing any external marks of violence. I do not think the injuries could have been inflicted on the previous Wednesday. There is no statement in the medical evidence of inflammation having occurred, which I should have expected had the injury been inflicted on the Wednesday. Had there been inflammation the pulse would have been affected. The respiration also would have been extremely difficult—that is, supposing the patient could so long have survived such injuries."

Cross-examined.—"I do not judge solely from the absence of evidence of inflammation in forming my opinion that the injuries could not have been received on Wednesday, but that is one of my reasons. A man might have his sternum and eleven ribs broken without any external ecchymosis. That might be, though the injury was done by kicks or blows, if he had his clothes on. I do not think that blows with the fist alone could have done all this mischief. I differ from the other medical witnesses in saying that these injuries must necessarily have been accompanied with ecchymosis, and that the injuries might have been inflicted on Wednesday. A man with one or two ribs broken might go on a day or two without exhibiting much inconvenience, but not without any. His being a lunatic would make no material difference. I differ from Dr. Tucker in thinking that it would. I have known cases where lunatics have injured themselves and have made no complaint. Among lunatics there are occasional instances of insensibility to pain, but in such cases there are other symptoms. There are other symptoms in this case. My impression is that the fractured ribs and sternum would have caused death immediately. Maniacs sometimes hurt themselves and do not complain."

Mr. Lewis.—"If the ribs had been fractured on the Wednesday, would that have caused death on the Saturday?"

Witness.—"I think it would have caused death at once, and he certainly would not have lived till Saturday without exhibiting very distressing symptoms, which could not have been masked."

Mr. Tyerman and Dr. Tucker, (one of the assistant medical officers of the Colney Hatch Asylum,) also gave evidence at the trial. Mr. Tyerman stated the opinions we have already ascribed to him, and on cross-examination he said:—

"The deceased was paralysed when he was admitted to the asylum, and his condition in this respect would render him less sensible to pain, and in some cases he believed it would occasion complete insensibility to pain. Patients afflicted with this form of insanity would sometimes attempt to walk about with broken limbs. He had seen several instances of this kind during his experience at Colney Hatch. In one case a patient who had fractured his knee-cap walked about without any apparent pain very shortly afterwards. In another instance, after a patient died, it was found that several of his ribs were fractured, and on the morning of the day of his death he said that he was quite well. He heard that this patient had sustained the injury in question by falling over his bed or over a chair. Cases of this sort were constantly occurring in lunatic asylums and other places. If there had been such a kicking and stamping upon the deceased as had been spoken to by the witnesses Clark and Varney, he was of opinion there must have been some external marks, but none such were visible upon the body of the deceased.* It was his opinion that many of the injuries sustained by the deceased might have been caused by the struggle with Gann and the blows he received from him on the Wednesday before his death. He had known Clark and Varney for some time, and

* It is not easy to conceive that Swift could have been pounded about the chest and stamped upon without being bruised, when we know that a blow upon the abdomen had occasioned an extensive bruise. It may be conceived, however, and it is not improbable, that if the attendant, a weighty man, fell heavily across Swift's chest during the struggle, the ribs and sternum might have snapped. Severe fractures of the ribs under such circumstances may, we believe, be found in the records of many asylums.

they had been under his care, and he was of opinion that no reliance could be placed upon any statement made by them, and if he were upon a jury he certainly should not give any credit to them. They were both subject to nervous delusions, and Varney had certainly a homicidal tendency, and a desire to conspire to destroy the lives of others."

Re-examined.—"There were no external marks to indicate by what means the ribs and sternum became fractured."

"Dr Tucker deposed that he saw the deceased between twelve and one o'clock on the morning of the day on which he died, and felt his pulse, which was moderately good. He made no complaint, and there was nothing to indicate that he had anything the matter with him."

Cross-examined.—"The deceased made no complaint of violence being used to him on Friday evening. He had heard the description given by the witnesses Clark and Varney, and he was of opinion that if the deceased had been kicked and stamped upon in the way they represented, for nearly an hour, there must have been some external marks of violence. Witness had had a good deal of experience of insane patients who were at the same time paralytic, and he was aware of several remarkable instances of their apparent insensibility to pain. In one case, where a patient had broken both the bones of his right leg, he pulled off the splints and tried to walk about on the stump, without exhibiting any appearance of pain. He considered it quite possible that all the injuries might have been caused on the Wednesday, and the deceased might not have exhibited any indication of the injury during the interval between that day and his death."

After a brief consultation the jury found both prisoners *Not Guilty.*

In a medico-legal point of view, this case is one of remarkable interest. Three of the most distinguished surgeons in London enter the witness-box, and upon oath, in a charge of manslaughter, assert, (1) that the fact of lunacy would make no material difference in the judgment to be formed of the consequences of certain severe physical injuries; and (2) that those injuries were of so grave a nature that it was "impossible" that the person subjected to them could have lived more than a very brief period after their infliction, or, supposing him to have lived for a longer or shorter time, that he would have exhibited manifest symptoms of serious disorder.

We shall not stay to question how it comes to pass that these gentlemen entirely ignore the recorded experience, and the facts upon which that experience has been based, of all the physicians of celebrity who have had charge of lunatics, from the days of Esquirol to our own time, on the not uncommon occurrence among the insane of a greater or less degree of insensibility to pain (not indifference to, as Mr. Luke and Mr. Partridge worded it at the Central Criminal Court) which has altogether masked, or singularly modified, the symptoms of the most serious surgical injuries. We can merely note, without seeking to explain, the singular fact that the opinion of the distinguished senior surgeon to St. Luke's Hospital upon this subject clashes with that of probably every existing practitioner in lunacy. We shall simply cite one or two facts bearing upon the assertion, that the amount of injury inflicted upon Swift's chest was such that it was impossible that he could have lived even for a short period, without showing it manifestly in his breathing or otherwise.

A. B., a tall, stout, middle-aged man, with a dark complexion, and a wood carver by trade, was admitted into the —— asylum. He had been insane two years before, but was said to have recovered, and had, on his admission, been ailing only six weeks, this latter statement being somewhat doubtful. He was quite paralytic, spoke in a loud blustering tone, and in the deliberate manner of a general paralytic. He had the usual class of delusions found in such cases—enormous wealth, and so forth. He would sleep about half the night, and when awake was rather noisy. He ate heartily, even greedily, and with the exception of the symptoms stated, seemed in excellent health. He was occasionally much excited during the day, but was readily pacified. Unless carefully watched he was apt to fall, as his legs afforded him but little support.

As this patient was so noisy and loquacious he was not examined physically, in the hope that the excitement would shortly subside, but he died rather suddenly on the thirteenth day after admission.

An examination of the body disclosed ten ribs fractured, six on the left side (from the fourth downwards) and four (from the sixth) on the right. The fractures occurred in a line extending from the junction of the middle and anterior thirds of the highest broken rib to the anterior extremity of the first false ribs. Several of the ribs were broken in three places. In these ribs one point of fracture was found to be converted into a false joint, a second to have a collection of pus, and a third to have callus around it, but not of any recent date. Fluid was found in both pleural cavities, the left containing about a gallon, the lower two-thirds of the lung on the same side being quite carnified. The right lung was healthy, the heart slightly enlarged, its walls being very thin. A few hours before his death this man had been exhibiting his "very fine voice," as he called it, running up and down the scale in a very powerful but by no means correct manner. He appeared to be quite unconscious that any thing was the matter with him, and made no complaint whatever of pain and uneasiness, and expressed himself well satisfied with the treatment he received in the asylum. He was continually exclaiming how well he was, and nothing, either in his movements or in his breathing, had led to any suspicion that the walls of his chest had been seriously injured. An inquiry led to the conclusion that the injuries had been most probably occasioned by frequent falls, several of them across a heavy iron fender, which the man had suffered previous to his entry into the asylum. He was a heavy man, but after each fall he had contrived to scramble on his feet again, and invariably professed, in the manner of patients of this class, that he had not hurt himself.

On comparing this case with that of Swift's, it will be admitted that, if anything, the injuries to the chest in the former instance were of a more serious character than in the latter, and yet we find them completely masked until the death of the patient, by insensibility to pain.

M——, an incoherent and extremely restless lunatic, moved about as usual, made no complaint of pain or injury, and ate heartily up to the time of his death. On examining the body, six ribs were found broken on the right side and one on the left. No suspicions of these fractures had existed before death, although special attention had been drawn to the right side by the appearance of a swelling, which subsequently proved to contain pus, consequent upon the fractures. From the appearance of the fractured bones and from a rigid inquiry, there was not the slightest ground for concluding that the injuries were of less than twelve days' standing.

If then a greater or less degree of insensibility to pain has such an important effect in modifying or masking the symptoms ordinarily arising from serious physical injury to the thorax, it might be presumed—if there were not, indeed, numerous cases to bear witness—that the same condition would equally modify or mask the symptoms arising from injury in other parts of the frame. It is not too much to assume that it would not exercise an insignificant influence in masking injuries to the substance of the liver. But surmising that the hæmorrhage from the injured liver was the immediate cause of death in Swift's case, it is all-important to know if this would necessarily occur contemporaneously with the infliction of the injury. This question, so far as our information extends, does not admit of a positive reply. We know, however, that serious injuries of the liver are not of *necessity* followed by hæmorrhage at all;* also that when hæmorrhage does occur from a wounded liver, it may be recurrent.†

A case recorded by Dr. Walter Fergus, and published in the 31st volume of the Medico-Chirurgical Society's *Transactions* (p. 47), is exceedingly instructive. The following is a brief summary of this case:—

William Fisher, æt. 17, was standing on the shaft of a cart, when the horse moved; he fell, and the wheel passed over the belly. He hardly complained, but said that there was a slight pain in the right side. The shock was very trifling, and no marks of violence were perceptible, and there was no tenderness on pressure. The pulse was thought to be a little accelerated. The night following the accident great pain supervened, which yielded to bleeding and opium. On the subsequent day he complained of pain only when he lay on his back. The next day (Monday) he had slight pain; on Wednesday his appetite returned, and he left his bed; on Friday he talked about leaving the hospital, but this was not agreed to, the pulse being sharp and the skin hot. The same day he was attacked with symptoms of acute peritonitis, and on the following Sunday he died, the ninth day after the accident.

"On cutting through the abdominal parietes there was an immense gush of a dark liquor, having precisely the colour and odour of bile; in fact, it appeared only to differ from that fluid in being more liquid. On laying open the cavity of the abdomen, the intestines were found roughened as in the first stage of acute peritonitis. The liver was seen with a laceration, extending in the direction taken by the broad ligament, quite through its substance, and to a depth from the thin edge of two inches and a half; another laceration extended about two-thirds of the length of the convex surface in a transverse direction; this was of a comparatively slight depth, and was in a state advancing towards reparation." The gall-bladder was also ruptured. No traces of hæmorrhage are recorded.

It is manifest, therefore, from this case and the cases we have referred to, that, in a medico-legal point of view, it is requisite to speak in the most cautious manner of the immediate consequences of rupture of the liver, even in the instance of persons suffering from no form of mental disorder.

We therefore feel fully justified in questioning the opinions of the three eminent surgeons examined for the prosecution, (1) as to the immateriality of lunacy in a medico-legal inquiry concerning surgical injuries; and (2) as to the necessity of a rapid fatal

---

* See case quoted from the *Mémoires de l'Académie Royale de Médecine* for 1705, by Guthrie, in his Lectures on *Wounds of the Abdomen and Pelvis;* Lect. i.

† See Paroisse, *Opuscules de Chirurgie.*

termination to those injuries, and the impossibility of their existing without giving rise to manifest symptoms during life.

It is not to be forgotten, however, that the opinions of Mr. Tyerman and Dr. Tucker on Swift's case were entirely based upon the assumption that he was to a greater or less extent insensible to pain. This supposition, in the absence of all actual experiment, could only have been warranted by the total failure of evidence calculated to throw a clearer and more decisive light upon the case. But, even in the absence of this evidence, was the assumption warranted? Is a diminution of sensitiveness to painful impressions observed with sufficient frequency among lunatics to justify the assumption in such a case?

On this point we have the recent positive assertions, derived from experiment, of Dr. A. Brierre de Boismont, one of the most distinguished alienist physicians in Europe. He states that—"Anæsthesia has been met with many times in insanity on careful examination. It is common in the second and third stage of the disease. *For a long time we have experimented on our paralytics* [the class of cases, be it borne in mind, to which Swift belonged] *and in nearly every case we have noted diminution of cutaneous sensibility; in some instances, indeed, there was complete loss.*"*

It cannot be needful that we should add many other remarks upon this important and highly interesting medico-legal case. From a careful review of the whole of the circumstances, we think that the conclusion must be obviously in favour of the opinions expressed by the medical superintendent of the male department in Colney Hatch. That these are justified by the evidence adduced, and that they are those which best meet the difficulties of the case, we fully believe; and we are much gratified to think that, medically as well as legally, there is no necessity to suppose that a brutal homicide has been perpetrated in the wards of our largest public asylum.

In conclusion, we would remark that this case of Swift's indicates the importance of noting, from time to time, the actual degree of cutaneous sensibility possessed by patients in asylums.

---

* "Medico-Legal Studies on the Perversion of the Moral and Affective Faculties in the Precursory Period of General Paralysis"—*Annales d'Hygiène Publique et de Médecine Legale*, Octobre, 1860. See also p. 60 of the present number of this Journal. We would, in further illustration of this subject, direct attention to a remarkably interesting paper by Dr. Auzouy, "Des Troubles fonctionnels de la Peau et de l'Action de l'Electricité chez les Alienes," to be found in the October number of the *Annales Médico-Psychologiques* for 1859. See also a translation of the portion of this paper, relating to cutaneous sensibility, in the *Journal of Psychological Medicine*, vol. xiii. p. 68. A remark of Dr. Auzouy's may be reproduced here with advantage. "M. Beau," he states, "has established a distinction as exact as ingenious between anæsthesia of pain, or *analgesia*, and anæsthesia of touch; he justly observes that tactile anæsthesia necessarily begets anæsthesia of pain, whilst the contrary does not hold. In fact, analgesia exists most commonly among the insane, the tactile sensibility not having disappeared."

